# Stability of Chitosan—A Challenge for Pharmaceutical and Biomedical Applications

**DOI:** 10.3390/md13041819

**Published:** 2015-04-01

**Authors:** Emilia Szymańska, Katarzyna Winnicka

**Affiliations:** Department of Pharmaceutical Technology, Faculty of Pharmacy, Medical University of Białystok, Mickiewicza 2c, Białystok 15-222, Poland; E-Mail: kwin@umb.edu.pl

**Keywords:** chitosan, drug delivery system, long-term stability, acidic hydrolysis, thermal degradation, storage conditions

## Abstract

Chitosan—one of the natural multifunctional polymers—due to its unique and versatile biological properties is regarded as a useful compound in medical and pharmaceutical technology. Recently, considerable research effort has been made in order to develop safe and efficient chitosan products. However, the problem of poor stability of chitosan-based systems restricts its practical applicability; thus, it has become a great challenge to establish sufficient shelf-life for chitosan formulations. Improved stability can be assessed by controlling the environmental factors, manipulating processing conditions (e.g., temperature), introducing a proper stabilizing compound, developing chitosan blends with another polymer, or modifying the chitosan structure using chemical or ionic agents. This review covers the influence of internal, environmental, and processing factors on the long-term stability of chitosan products. The aim of this paper is also to highlight the latest developments which enable the physicochemical properties of chitosan-based applications to be preserved upon storage.

## 1. Introduction

Nowadays, besides novel drug molecules discovery processes, the development of multifunctional drug delivery systems has become a current and attractive concept in pharmaceutical technology. Carbohydrate-based vehicles with a capability of reducing dosing frequency, improving drug pharmacological activity and delivering drugs at the specified site appear to be promising as pharmaceutical drug carriers [1,2,3]. Among various carbohydrate polymers, chitosan—a natural multifunctional polysaccharide—due to its biocompatibility, biodegradability, and mucoadhesiveness has been extensively studied for a number of biomedical and pharmaceutical applications, including prolonged or controlled release drug delivery systems [4], wound dressings [5], blood anticoagulants [6], cartilage and bone tissue engineering scaffolds [7,8], and space filling implants [9]. Chitosan is a polycationic copolymer, consisting of glucosamine and *N*-acetylglucosamine units, obtained by deacetylation of chitin derived from the exoskeleton of crustaceans, insects, or fungi [10,11]. It is available in a wide range of degrees of deacetylation and molecular weight, which are also the main factors influencing the nature and quality of the polymer. Chitosan—as an abundantly accessible and inexpensive biomaterial—can be easily formed into diverse semi-solid and solid structures under mild conditions. It is soluble only in diluted inorganic and organic acids with a pH lower than chitosan pK_a_ (about 6.3), forming a non-Newtonian, shear-thinning fluid [12]. At low pH, the free amino groups are protonated causing electrostatic repulsion between the polymer’s chains and thus enabling polymer solvation. Chitosan possesses good mucoadhesive properties resulting from the cationic behavior and the presence of free hydroxyl and amino groups allowing the polymer to interact with mucin by hydrogen and electrostatic bonding. Hence, it is regarded as a suitable excipient to prepare buccal [13], nasal [14], ocular [15] and vaginal dosage forms [16]. In addition, chitosan is reported to show penetration enhancement properties by improving active agent transport through the epithelium layer containing tight junctions [17]. Due to its mucoadhesiveness and ability to cross epithelial barriers, chitosan has been widely studied as a vaccine adjuvant or co-adjuvant as it was shown to enhance bioavailability and immunogenicity of antigens after oral, nasal, or subcutaneous administration [18,19,20]. Superior hemostatic efficacy of chitosan through platelets activation and thrombin generation was also displayed [6] enabling its application in wound dressings [5]. The polymer is also considered as a promising candidate in obesity and hypercholesterolemia treatment as it is able to combine bile acids in the digestive tract and in consequence increase their excretion [21]. Numerous data have drawn attention to the use of chitosan as an antifungal and antibacterial agent [22,23,24]. Furthermore, chitosan has been recently employed as an adjunctive for an antimicrobial drug in order to increase its pharmacological activity [25,26]. Examples of chitosan-based delivery systems and biomedical devices are shown in Table 1.

**Figure 1:**
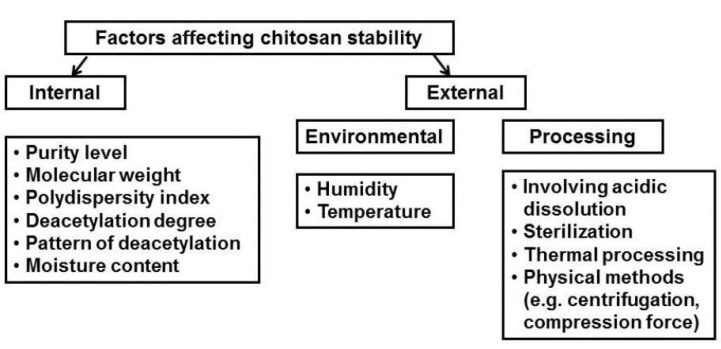
Factors affecting stability of chitosan-based products.

Despite the fact that chitosan is a unique and versatile compound, widely used in the pharmaceutical and biomedical fields, there are hardly any available pharmaceutical products based on chitosan (only hemostatic dressings, preparations for wound-healing and nutraceutical products exist) (Table 2). This might be a result of the strong hygroscopic nature of chitosan and the fact that chitosan material extracted from various sources differs significantly in terms of its molecular weight and molecular weight distribution, degree of deacetylation, and purity level. Additionally, the high susceptibility of chitosan to environmental factors and processing conditions (such as heating or freezing) can impose stress on its structure and cause polymer degradation (Figure 1).

**Table 1:** Examples of chitosan (CS)-based drug delivery systems and biomedical devices.

Material	Active Substance	Dosage Form	Biomedical or Pharmaceutical Application	References
Composition of unmodified CS, ethyl cellulose and butylphtalate	Buspirone hydrochloride	Sustained release lyophilized sponges	Buccal treatment of anxiety	Kassem *et al.*, 2012 [13]
CS/xanthan polyelectrolyte complex	Promethazine hydrochloride	Mucoadhesive inserts	Nasal treatment of migraine	Dehghan *et al.*, 2014 [14]
Unmodified CS	Bimatoprost	Sustained release inserts	Ophthalmic treatment of glaucoma	Franca *et al.*, 2014 [15]
Unmodified CS and CS crosslinked with β-glycerophosphate	Clotrimazole	Prolonged release microgranules, tablets and hydrogel	Vaginal treatment of candidiasis	Szymańska *et al*., 2012 [4] Szymańska *et al.*, 2014 [16,25]
Unmodified CS	Chloramphenicol	Sustained-release liposomal hydrogel	Topical, wound therapy	Hurler *et al.*, 2012 [5]
Unmodified CS	Metronidazole	Hydrogel	Periodontal therapy	Akncbay *et al.*, 2007 [22]
*N*-trimethyl CS	Ovalbumin	Nanoconjugates	Nasal and intradermal vaccination	Slűtter *et al.*, 2010 [18]
*N*-trimethyl CS crosslinked with tripolyphosphate		Nanoparticles		Bal *et al.*, 2012 [19]
CS crosslikned with glucose-1-phosphate	Diclofenac potassium	*In situ* forming hydrogel	Injectable	Supper *et al*., 2014 [27]
Composition of CS crosslinked with β-glycerophosphate and glucosamine	Articular chondrocytes	*In situ* forming hydrogel	Cartilage and bone tissue engineering	Hoemann *et al*., 2005 [9]
CS crosslinked with citric acid	Cisplatin	Microspheres	Dry powder inhalation system for lung cancer	Singh *et al*., 2012 [28]
Complexation of CS and dextran sulfate	Insulin	Nanoparticles	Oral delivery for insulin/diabetes therapy	Sarmento *et al.*, 2006 [29]
CS/alginate composite	Fucoidan	Freeze-dried scaffold	Bone tissue engineering	Venkatesan *et al.*, 2014 [8]

**Table 2:** Examples of commercial medical devices and oral nutraceuticals with chitosan (CS).

Product	Material	Usage/Application	Manufacturer
**Wound-healing and hemostatic products**			
Chitodine^®^	CS powder with adsorbed elementary iodine	Disinfection of wounded skin, surgical dressing	International Medical Services
ChitoPack C^®^	Cotton-like CS	Regeneration and reconstruction of body tissue, subcutaneous tissue and skin	Eisai Co.
Celox^TM^	Gauze and granules with CS	Control of bleeding from non-cavitary grain wounds	MedTrade
ChitoFlex^®^	CS acetate sponge		HemCon Medical Technologies INC.
HemCon^®^ Bandage Pro HemCon^®^ Strip First Aid	Freeze-dried CS acetate salt		
PosiSep^®^	*N,O*-carboxymethyl CS sponge	Intranasal hemostatic splint for patients undergoing nasal/sinus surgery	Hemostatis LLC.
Syvek Excel™	Lyophilized three-dimensional CS fibers	Rapid control of bleeding for anticoagulated patients	Marine Polymer Technologies Inc.
Clo-Sur^®^ PAD	Non-woven seal with a soluble CS	Control of moderate to severe bleeding	Scion Cardio-vascular
ChitoSeal^®^	Soluble chitosan salt		Abbott Vascular Devices
TraumaStat^®^	Porous polyethylene fibers filled with silica, coated with CS (ChitoClear^®^)		Ore-Medix
Tegasorb^®^	CS particles		Tesla-Pharma
Vulnosorb^®^	Composition of microcrystalline CS with fibrinogenic tissue glue		3M
**Nutraceutical products**			
Slim Med™	Non-animal CS	Prevention and treatment of overweight	KitoZyme S.A.
KiOcardio™	Non-animal CS	Maintenance of normal blood cholesterol level	KitoZyme S.A.
LipoSan Ultra^®^	Composition of CS (ChitoClear^®^) and succinic acid	Binding dietary fat and reducing its absorption in the intestine	Primex
Liposorb™	CS extracted from squid	Preventing irritable bowel syndrome; Binding dietary fat and reducing its absorption in the intestine	Good Health

**Figure 2:**
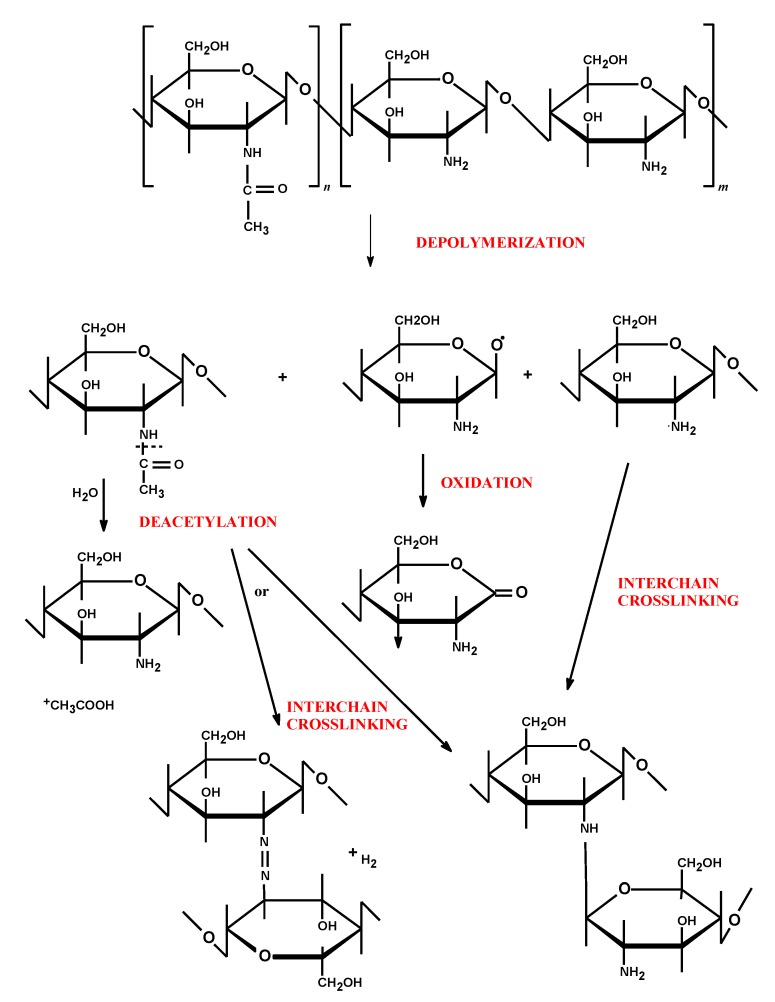
Possible degradation mechanisms of chitosan’s structure (adapted from [32] with modifications).

Chitosan as a natural biodegradable biopolymer undergoes enzymatic transformation to basic, non-toxic components. Chitosan is degraded *in vivo* by several enzymes, mainly by lysozyme—a non-specific protease present in all mammalian tissues—producing non-toxic oligosaccharides which can be then excreted or incorporated to glycosoaminoglycans and glycoproteins [30]. *In vitro* degradations of chitosan via oxidation, chemical, or enzymatic hydrolysis reactions are commonly used methods for the preparation of low molecular chitosan under controlled conditions [31]. The molecular weight, polydispersity, deacetylation degree, purity level and moisture content play a crucial role in determining the mechanism and the speed of polymer degradation. Regardless of the mode of degradation, the process usually begins with random splitting of β-1,4-glycosidic bonds (depolymerization) followed by *N*-acetyl linkage (deacetylation) (Figure 2). As a consequence, a decrease in average molecular weight and an increase in deacetylation degree are observed. Simultaneously with chitosan chain scission, cleavage and/or destruction of its functional groups (amino, carbonyl, amido, and hydroxyl) occur. In addition, chitosan depolymerization may lead to formation of free radicals which induce oxidation processes [32]. Strong intermolecular interactions between formed fragments of chitosan (interchain crosslinking) alter the polymer structure, thus leading to the irreversible loss of its physicochemical properties. Despite the fact, that numerous data have drawn attention to the chitosan-based applications in the pharmaceutical and biomedical field, only a limited number of studies and review articles have been devoted to long-term stability studies on chitosan-based assemblies [33,34,35,36,37].

This review considers the issue of chitosan’s degradation mechanism and provides insight into internal, environmental and technological factors affecting the storage stability of chitosan-based systems. Furthermore, the focus of this paper is to describe different strategies and recent advancements implemented to preserve physicochemical properties of chitosan applications upon storage such as addition of stabilizer during the preparation process, formation of polymer blends, and use of ionic or chemical crosslinkers. Due to the wide range of this topic, improvement of the long-term stability of chitosan products by modification of chitosan’s structure via grafting functional groups will be not considered in this review. As a high risk of uncontrolled chitosan decomposition arises from inappropriate storage conditions, the influence of temperature and humidity upon storage stability studies is also highlighted.

## 2. Influence of Internal Factors on Chitosan’s Stability

### 2.1. Purity Level

Although chitosan-based applications have been widely investigated in the biomedical field, there is still a lack of worldwide clear and definite requirements for chitosan as a pharmaceutical excipient. Monographs relating to chitosan and chitosan hydrochloride were first introduced into the European Pharmacopeia 6.0 and United States Pharmacopeia 34-NF 29th edition, respectively [38,39]. The chitosan pharmacopeial properties are summarized in Table 3. Chitosan is commercially available in various grades of purity, molecular weight, and degree of deacetylation. The wide range of chitosan sources and variety of its manufacturing processes lead to great differences in the quality and properties of chitosan products, which as a result might deviate from the pharmacopeial recommendations. Furthermore, the specification data provided by the chitosan suppliers are often incomplete, which may be misleading for pharmaceutical technologists. Although chitosan preparation involves basic purification methods like demineralization and deproteinization, chitosan material may contain some impurities, such as ash, heavy metals, or proteins. The purity level of chitosan is a factor which affects not only the biological properties like immunogenicity or biodegradability, but also has a profound effect on its solubility and stability. High ash and residual proteins content may cause difficulties in chitosan dissolution and impede preparation of chitosan-based drug delivery systems. On the other hand, microbiological contamination of the polymer may enhance chitosan degradation via enzymatic hydrolysis. Therefore, chitosan material should be of high purity and be free of contaminants (including the level of endotoxins where relevant).

**Table 3:** Chitosan and chitosan hydrochloride properties recommended by the European Pharmacopeia 6.0 and the United States Pharmacopeia 34-NF 29 [38,39].

Parameter	Acceptance Criteria	
	Eur. Ph. 6.0 *Chitosan hydrochloride*	USP 34-NF 29 *Chitosan*
Appearance of solid product	White or almost white fine powder	n.d.
Degree of deacetylation	70.0%–95.0%	70.0%–95.0%
Distribution of molecular weight *	n.d.	0.85–1.15
pH of 1% (g/mL) solution	4.0–6.0	n.d.
Loss on drying *	n.d.	≤5%
Insolubles/Impurities	≤0.5%	≤1.0%
Heavy metal	≤40 ppm	≤10 ppm
Iron	n.d.	≤10 ppm
Sulphated ash *	≤1%	n.d.
Protein	n.d.	≤0.2%
Microbiological contamination	n.d.	Absence of *Pseudomonas aeruginosa* and *Staphylococcus aureus*
Aerobic microbials *	n.d.	10^3^ cfu
Molds and yeasts *	n.d.	10^2^ cfu

*: determined on 1.0 g sample; n.d.—not determined.

### 2.2. Molecular Weight and Molecular Weight Distribution (Polydispersity)

Chitosan is primarily characterized by its molecular weight, which is responsible for a number of its physicochemical and biological properties such as hydrophilicity, viscosity, water-uptake ability, biodegradability, and mucoadhesion [10,40]. The molecular weight (M_W_) is expressed as an average of all the molecules present in the sample. With regard to the initial source material and the type of preparation method, the M_W_ of commercial chitosan varies between 10–100,000 kDa. The average molecular weight may be estimated by a number of techniques, such as osmometry, light scattering, NMR, viscometric assay of the polymer’s intrinsic viscosity, or chromatographic techniques (size exclusion chromatography, gel permeation chromatography). All of these measurements display experimental difficulties and should be properly validated. Additionally, the M_W_ of chitosan samples may differ depending on the applied technique which can be misleading for technologists and complicate direct comparison between polymer materials obtained from different manufactures. The process of deacetylation may decrease the polymer M_W_ [41]. In order to ensure chitosan’s uniformity and proper functionality in the final product, the molecular weight distribution (polydispersity index, PDI) should be determined [39,42]. PDI refers to the ratio of M_W_ to a number of average molecular weights (M_N_) and a value between 0.85 and 1.15 is considered as having good polymer homogeneity [39]. Generally, high molecular weight chitosan is regarded as more stable. The M_W_ was found to affect the thermal stability of the polymer [32]. In addition, a number of factors, including strong acids, elevated temperature, mechanical shearing, or irradiation may influence the chitosan M_W._ For instance, physical methods—high pressure homogenization, extensive shearing, or centrifugation—frequently used for preparation of biomedical chitosan devices, were noticed to decrease the polymer M_W_ and were responsible for the fluctuations of the PDI [43]. It should be also noted that the compression force during tablet preparation is responsible for heat generation and might influence the chitosan M_W_ distribution [44].

### 2.3. Degree of Deacetylation and the Pattern of Deacetylation

The degree of deacetylation (DD) is the ratio of glucosamine to *N*-acetylglucosamine units, while distribution of these groups along the polymer chain is described as the pattern of deacetylation (P_A_). The degree of deacetylation of commercial chitosan is controlled by modifying the time and temperature of the de-*N*-acetylation process [41] and according to the pharmacopeial specifications, the parameter ranges from 70% to 95% [38,39]. Furthermore, with regard to deacetylation conditions, chitosan may show a characteristic P_A_ that varies from block to a random type [45]. It should be particularly important to accurately define the chitosan DD and P_A_, as they are—similarly to M_W_—crucial factors determining its physicochemical behavior and biological functionality. Recently, considerable research effort has been made to investigate the effect of DD on the potency and the rate of chitosan’s degradation. Interestingly, polymer with low DD was found to induce an acute inflammatory response as a result of the fast rate of degradation, while chitosan with high DD caused minimal inflammation [30]. This is in agreement with observations made by Zhang *et al.*, who showed that chitosan with high DD possessed lower affinity to enzymes *in vitro* [46]. Moreover, the P_A_ was noticed to influence biodegradability, since homogenous distribution of acetylated groups (random type of P_A_) resulted in a lower rate of enzymatic degradation [47]. The contamination level of chitosan material—which correlates with the DD—may also have an impact on the polymer immunogenic behavior. Yuan *et al*., showed that the higher the DD, the higher the purity grade observed in the polymer sample [48]. Thus, careful selection of chitosan with proper DD should be of great interest, especially with regard to parenteral chitosan-based formulations.

Several studies revealed that chitosan DD affects both hydrolytic and thermal behavior of the polymer products [32,49,50,51]. It was found that the more extensive de-*N*-acetylated chitosan sample, the slower the rate of acidic hydrolysis observed during storage [49]. This phenomenon was explained by the fact that chitosan with higher DD has a less porous structure and lower water-uptake ability, which limits the rate of the degradation process in acidic environment. On the contrary, a slower rate of chitosan thermal depolymerization may be a result of interchain crosslinking between free amino groups, which exerts a stabilizing effect on the polymer’s structure [32,50,51]. Nevertheless, chitosan with high DD was also shown to be more susceptible to photodegradation [52]. The P_A_ has a significant impact on the charge density, which in turn affects the solubility behavior of chitosan with the same M_W_ and DD [45]. For instance, chitosan with a block pattern of acetylated and deacetylated units was shown to aggregate in acidic environment impeding its dissolution process [53].

### 2.4. Moisture Content

Chitosan is hygroscopic in nature, having a greater capability to form hydrogen bonding (formed with both hydroxyl and amino groups) with water compared to chitin [54]. The amount of absorbed water depends on the initial moisture content as well as on the storage conditions, especially the environmental temperature and relative humidity. Rege *et al*., found that the moisture level of chitosan powder ranged from 7% to 11% (w/w) and was independent of the polymer DD or M_W_ [55]. However, Mucha *et al*., noticed that the water-uptake ability of chitosan films decreased on increasing their DD [56]. The presence of absorbed water plays a considerable role especially in solid chitosan-based formulations, affecting the flow properties and compressibility of the powders’ or tablets’ tensile strength. It was reported that moisture content up to 6% (w/w) may improve particle binding during compression as a result of formation of hydrogen bonds between the particles [57]. However, fluctuations in moisture levels of chitosan material upon storage may change the physicochemical and mechanical properties of chitosan-based systems. Studies conducted by No *et al*., revealed that although the level of absorbed water in chitosan powder rose during storage, a decline in water binding capacity was observed [58]. Viljoen *et al*. showed that six-month storage of chitosan tablets caused a dehydration of the polymer, which resulted in a decrease in crushing strength followed by an increase in friability and disintegration time. In addition, the higher the water content in the chitosan structure, the faster and more pronounced was the damage of the polymer (via hydrolysis reactions) observed [59].

As initial moisture level and strong hygroscopic behavior may limit chitosan’s applicability, the water content in chitosan material should be measured and optimized prior to preformulation studies and carefully controlled upon storage. Among various methods of moisture content determinations in solid forms, loss on drying technique is a simple and quick method, in which a material sample is weighed, heated in an oven, and reweighed after cooling [39]. The swelling index test is another commonly used method, which helps to investigate how the chitosan water-uptake ability changes upon long-term storage. The measurements, which can be applied for both semi-solid and solid formulations, consist in placing an accurately weighed sample in an acceptance medium (e.g., suitable body fluid simulant), usually at 37 °C. It is important to carefully select the type of the medium prior to the swelling studies, e.g., pure water usage should be excluded for experiments with unmodified chitosan-based formulations because of the impact of ionic strength on the chitosan’s viscosity and swelling behavior and its poor solubility in water pH. At a predetermined time interval, the formulations are periodically weighed until a constant weight is obtained. The swelling ratio is then calculated using the following formula:

(1) $$[\mathrm{SR}]=\frac{W_{S}-W_{O}}{W_{O}}$$

where: SR—swelling ratio, *W*_O_—initial weight of dosage form, *W*_S_—weight of dosage form after swelling [60].

## 3. Influence of External Factors on Chitosan Stability

### 3.1. Environmental Factors

Chitosan is very sensitive to environmental conditions, hence it is recommended to store in closed containers at low temperatures (2–8 °C) [38]. In the preparation process of chitosan-based applications it is particularly important to establish the shelf-life of the product by conducting stability studies [27,59,61]. The purpose of the stability testing is to provide reliable evidence on how the quality of the chitosan product differs with time under the influence of environmental factors such as humidity and temperature. Type of stability studies (long-term, intermediate, or accelerated), storage conditions and frequency of testing should be selected with respect to the chitosan formulation properties [62]. The impact of the crucial environmental parameters—relative humidity and temperature—on the physicochemical properties of chitosan applications upon storage is presented below.

#### 3.1.1. Humidity

The presence and distribution of moisture in the chitosan material strongly depends on the ambient relative humidity (RH). For relative low humidity (below 40%), water transport in chitosan material was shown to follow a Fickian process, whereas at higher values of humidity, an anomalous diffusion kinetic was observed [63]. The analysis of chitosan water sorption carried out under ambient conditions (25 °C, 60% RH) showed that chitosan absorbed 14%–16% (w/w) of water (within 100 min) and the process rate was dependent on the polymer DD [56]. In high humidity conditions (RH > 60%), water molecules were found to penetrate more intensively through chitosan chains, thus the chitosan moisture content increased significantly [58,63]. The environmental moisture content is responsible for a plasticizing or swelling effect of solid or semi-solid polymer assemblies, respectively. Long-term storage at high RH may not only accelerate the ratio of chitosan hydrolytic damage, but also alter the polymer’s physicochemical and biological properties. Long-term stability studies revealed that chitosan tablets stored for six months at 70% RH possessed markedly lower mechanical properties compared to those kept at 60% RH [59]. Similar observations were made in the study on chitosan/amylose corn starch composite films which became mechanically weaker upon three-month storage at 40 °C/70% RH [64]. In the case of semi-solid chitosan applications, storing at high RH and changing its water-uptake ability alter the rate of drug release profile of the chitosan matrix. Kurek *et al*., noticed that the increase of ambient humidity from 0% to 75% resulted in a greater swelling of chitosan films, which was responsible for a greater and faster release of active compound from the chitosan carrier [65]. In addition, excessive hydration at high relative humidity could weaken the mucoadhesive properties of chitosan carriers as a result of “dilution” of functional groups available for adhesive interactions with mucin [66].

#### 3.1.2. Temperature

Apart from relative humidity, temperature is another variable which exerts an effect on the moisture content in chitosan-based systems. Exposure to elevated temperatures (40 °C) was found to cause a significant loss of moisture (dehydration of chitosan powder), which resulted in a decrease in hardness and mechanical tablet strength [59]. In addition, air temperature may affect the chitosan degradation ratio, especially in liquid and semi-solid products. Storage of chitosan solution, both at ambient and elevated temperatures, resulted in faster degradation of chitosan chains [49,67] and the rate of hydrolysis was found to follow first-order kinetics. Interestingly, no significant chain hydrolysis was noticed in the chitosan solution stored at 5 °C [67]. Furthermore, long-term stability studies established on chitosan/glucose 1-phosphate thermosensitive solution confirmed the necessity of their storage in a refrigerator (at 2–8 °C) [27].

### 3.2. Processing Factors

#### 3.2.1. Processing Involving Acidic Dissolution

Chitosan degradation via hydrolysis is a particular problem in pharmaceutical technology because dissolution of chitosan in diluted acids is a routine stage in the pharmaceutical technology of chitosan-based formulations. During hydrolysis, acid acts as a catalyst which splits the polymer chains. As a result, a decrease in average M_W_, viscosity, and weakness of mechanical properties is observed. It was reported that the rate of hydrolysis followed first-order kinetic and the main factors affecting this parameter were: DD, polymer concentration, type of acid and its concentration, treatment time, and temperature. There are several studies devoted to chitosan hydrolysis using several types of acids, namely acetic [34,67], formic [67], lactic [68], and hydrochloric [49]. Different acetic acid concentrations were found not to affect the degradation rate [67], whereas an accelerated rate of hydrolysis with increased concentration of hydrochloric acid was observed [49]. A faster rate of chain damage was noticed when chitosan with lower DD was used in the studies. This phenomenon can be explained by the fact that chitosan with low DD possesses a more porous structure and electrostatic repulsion between protonated amino groups is more pronounced thus promoting penetration of acid solution inside the polymer structure. Nevertheless, it should be pointed out that an increase in temperature (regardless of the type of acid used) is regarded as accelerating the degradation rate of the polymer [37]. Interestingly, studies accomplished by Nguyen *et al*., revealed that the ratio of chitosan decomposition in acetic acid solutions could be slowed down by storage at 5 °C [67].

A simple and commonly used assay for stability testing of chitosan dispersions upon storage is a viscometric measurement of its intrinsic viscosity [η] using the Mark-Houwink equation:

(2) $$[\text{η}]=k\times M_{w}^{\alpha }$$

where *M*_w_—the viscosity-average molecular weight, η—the intrinsic viscosity, *k* and α (Mark-Houwink exponent) are empirical constants describing the polymer conformation [37].

The intrinsic viscosity of chitosan describes the ability to form viscous solution (under specific solvent and temperature conditions) and is directly proportional to the polymer average M_W_. The Mark-Houwink exponent is suitable for indicating a specific chitosan conformation. When α = 0 the chitosan structure is referred to as a compact sphere, α = 0.5–0.8—random coil and α = 1.8—rigid coil. This exponent is useful when exploring alterations in polymer conformation with an increase in polymer chain length. The intrinsic viscosity is also a simple and quick method useful for determination of the average Mw of soluble macromolecules. This test requires calibration procedure development as the Mark-Houwink constants differ according to the type of solvent and temperature of measurement. Due to a lack of reference standards and validation data, this technique has not been incorporated into the pharmacopeias as an alternative method for the polymer average M_W_ measurements.

#### 3.2.2. Sterilization

Chitosan-based drug delivery dosage forms intended for ocular or parenteral administration, and those which contact with wounds, require high microbiological purity and have to be sterilized. Commonly used sterilization methods of pharmaceutical products include filter sterilization, saturated steam sterilization, exposure to dry heat and ethylene oxide, or γ-radiation [38,39]. These methods act either physically or chemically and may lead to irreversible alteration in both chitosan structure and its function. The previous studies reported that sterilization of chitosan gels by saturated steam caused chain scission of the polymer which resulted in 20%–50% decrease in viscosity and almost 30% loss of M_W_ [69]. Similarly, Toffey et al., found that autoclaving was not suitable to sterilize chitosan films prepared in acetic acid, because harsh conditions which had been employed in the process reduced its tensile strength and diminished polymer solubility [70]. Moreover, Lim et al., noticed that chitosan heated at 160 °C for 2 h became insoluble in acidic solution, which may be related to interchain crosslinking involving the amino groups [71]. In addition, the experiments conducted by Lim et al., and Yang et al., showed that both dry heat (160 °C for 2 h) and autoclave sterilization (under the pressure 100 kPa, at 105–125 °C for 15–30 min) caused darkening of chitosan dried powder to a yellow color [71,72]. The authors suggested that the colored products which appeared as a probable consequence of the Maillard reaction between the amino and carbonyl groups, should be carefully examined in terms of their biocompatibility and cytotoxicity. On the contrary, some researchers did not notice significant changes in the chemical structure of chitosan suggesting autoclaving as a suitable sterilization method for solid chitosan devices [72]. San Juan *et al*., found that the Mw of chitosan was unaltered after steam sterilization when the chitosan powder was dispersed in water prior to the autoclaving process [73].

Gamma irradiation—another potential sterilization technique—was found to cause significant main chain scissions of both powdered chitosan and its films, even when performed at −80 °C [72,74,75]. The studies also indicated a significant decrease in M_W_ followed with an increase in DD in a γ-radiation dose-dependent manner. In addition, chitosan films which had been exposed to irradiation, were shown to possess lower water sorption capacity [74] and higher value of tensile strength, probably due to polymer chain rearrangements [76]. Interestingly, several studies revealed that exposure to ethylene oxide (EO) caused relatively minor changes in the structure and physicochemical properties of chitosan dried powder or membranes, suggesting this method as the most appropriate for chitosan devices [72,76]. On the contrary, studies on the solid chitosan samples revealed that EO sterilization caused structural alterations in the polymer, irrespective of DD as a result of oxidation of its amine groups. Interestingly, observed chemical changes were restricted only to the polymer surface [77]. It should be also noted that chitosan products sterilized by ethylene oxide have to be quarantined prior to use in order to remove gas residues.

The influence of ultraviolet light (UV) radiation on chitosan films was also investigated [32,52]. Results of those studies displayed degradation of the polymer subjected to UV exposure mainly by formation of free radicals and destruction of polymer amino groups. The rate of degradation was more pronounced in the case of chitosan with higher DD [52].

To eliminate microbiological contamination and to guarantee a high purity level of heat-labile liquid chitosan formulations, filter sterilization could be applied. This quick and simple method appears not to influence the stability of chitosan-based products. However, filter sterilization has several obstacles resulting from the chitosan M_W_ and its concentration. For instance, highly viscous chitosan solutions are quite likely to clog the filter membrane and could not be sterilized by filtration. In addition, the type of the filter material should be carefully selected with regard to the solvent used for chitosan solution preparation (e.g., cellulose acetate or nylon membranes cannot be used for organic or/and acidic solutions).

Given these points, it is particularly important to investigate the effect of the sterilization process on the physicochemical properties and the end performance of chitosan material. Alternatively, preparation of chitosan-based formulations under aseptic conditions should be considered if the above mentioned sterilization methods failed. However, in such a situation application of ultrapure chitosan material is required.

#### 3.2.3. Thermal Processing

##### Heating

Heat is often employed in preparation of chitosan-based formulations. Exposure to elevated temperatures might change a number of polymer properties, including aqueous solubility, viscosity, and appearance [37,71]. Chitosan decomposition during heating has been widely established and the rate and degree of the polymer damage was found to accelerate with rising temperature and duration of heating [70,71,78]. Thermal degradation of the chitosan structure, measured using the thermogravimetric method (TGA), is a complex reaction involving two or even three degradation stages [79,80,81,82]. The first stage occurs at temperature 30–110 °C and is assigned to the evaporation of the residual water present in the polymer sample. The second thermal event—attributed to the polymer decomposition—is observed over a wide temperature range, from 180 to 340 °C. The differences in glass transition temperatures were explained as a result of different M_W_ of the investigated chitosan [70] and the glass transition temperatures of chitosan samples were found to shift to a higher value with an increase in its M_W_. Moreover, Diab *et al*., presented the third stage observed at 470 °C with a subsequent weight loss of the chitosan sample [80]. On the basis of these results, it can be stated that chitosan may be heated up to temperatures below its glass transition temperature without affecting its physicochemical properties. Apart from the sterilization process (which was described in the previous section), preparation of pharmaceutical carriers or biomedical devices with chitosan usually does not involve heating above 100 °C. However, the time of gentle heating necessary to dissolve chitosan in acidic solution should be carefully controlled as overheating of a chitosan sample might not only cause polymer discoloration but also—as a result of the depolymerization process—change its rheological properties and/or, paradoxically, slow down its rate of dissolution [70]. In addition, the loss of water as a consequence of thermal treatment is regarded as being responsible for lowering the glass transition temperature, which makes the polymer more sensitive to temperature and subsequently reduces its stability during storage [83]. It should be noted, that the presence of a drug, plasticizer or other additives in chitosan-based systems tends to decrease the polymer glass transition temperature [60].

Currently, spray drying technique is an advanced thermal method of chitosan-based micro- and nanoparticles preparation widely used in the pharmaceutical technology [84,85]. The spray drying is an uncomplicated single phase process, in which dry particles are obtained from a fluid state by evaporating the solvent. In order to obtain chitosan micro- or nanoparticles with the desirable properties, understanding of the process and careful adjustment of the spray-drying conditions (e.g. the inlet temperature) are required [86]. With regard to active substance and excipients used in chitosan-based particles preparation, the inlet temperature can vary between 120 and 170 °C [84,85,87,88,89]. Although the fluid containing chitosan is exposed to high temperature for a very short period of time, the influence of this parameter on the end performance and the properties of chitosan product cannot be excluded. The large surface area of the chitosan micro- or nanoparticles is particularly exposed to a heat stream during spray-drying and thus it is most at risk of thermal decomposition and alteration of the polymer’s physicochemical properties, especially its electrostatic charge. This might result in a higher content of hydrolysis products on the particles’ surface and their accelerated aggregation [89].

##### Lyophilization

Lyophilization (freeze-drying) is a well-established drying method in which frozen material is dried by sublimation of ice. Lyophilization has many applications, especially for micro- and nanoparticles technology with the advantage of preventing not only particles aggregation but also the escape of encapsulated drug. Freeze-drying is also considered as a feasible strategy to improve the physicochemical stability of colloidal systems, including chitosan-based microparticulate delivery products over extended time periods [87,90]. Hafner *et al*., established a freeze-drying process for melatonin-loaded lecithin/chitosan nanoparticles in order to improve their poor physicochemical stability in aqueous suspension [91]. After seven months storage, all lyophilisates remained in an amorphous state and the content of entrapped melatonin did not alter. Additionally, these nanoparticles were found to have re-dispersed easily with no particle aggregation after reconstitution. Nevertheless, lyophilization may impose stress on labile materials, such as unmodified chitosan and damage of the polymer can occur [92]. This is because chitosan undergoes strong inter- and intramolecular hydrogen bonding and hydrophobic interactions which might negatively affect physicochemical properties, such as viscosity, zeta potential, and water-uptake ability. In addition, too harsh removal of the residual water from chitosan material could result in destabilization of the polymer structure. Several reports consider the lyophilization process as an inappropriate drying method for chitosan-based formulations, even when modified chitosan was applied. The chitosan/polyol-phosphate thermogelling solutions after freeze-drying were found to be unable to maintain their viscoelastic properties [93]. In comparison to freshly prepared solutions, stored formulations were shown to possess higher viscosities, an increased gel strength, and shortened gelation time, which made them inconvenient as a ready-to-use product in pre-filled syringes [93]. In another study, Dehghan *et al*., investigated the stability of lyophilized chitosan/xanthan polyelectrolyte complex for nasal delivery under accelerated conditions [14]. After three-month storage, the physical appearance of the nasal inserts did not alter and the drug content was within limits. However, a significant increase (26%) in weight as a result of moisture uptake was noticed, which was responsible for the acceleration of the drug release rate. The authors did not provide the stability data under either refrigerated or ambient conditions, which could have given a detailed insight into the stability of chitosan-based lyophilisates.

## 4. Strategies to Improve the Stability of Chitosan-Based Products

**Figure 3:**
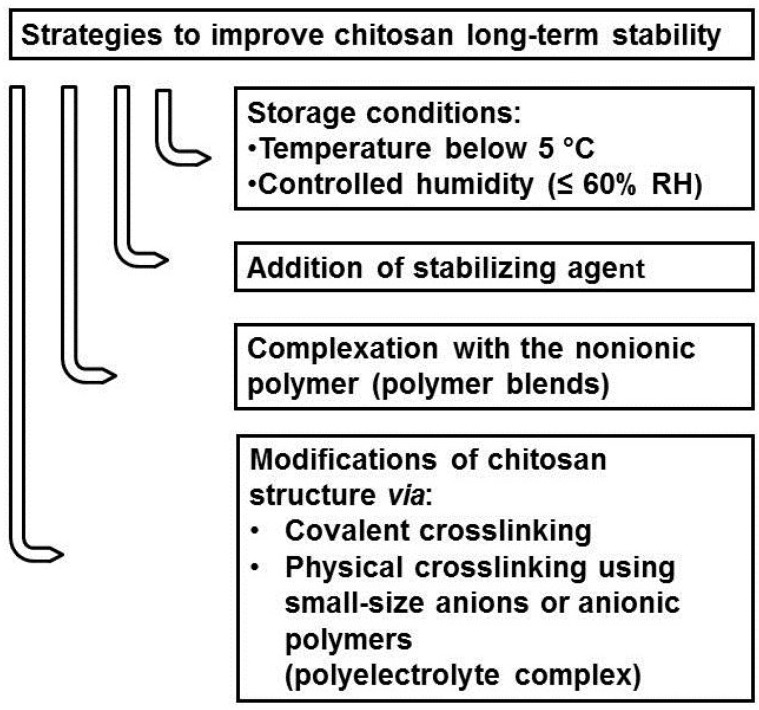
Strategies to improve the stability of chitosan-based products.

Over the last decades, chitosan has increasingly drawn attention as an attractive compound in the biomedical and pharmaceutical fields [4,5,6,7,8,47,69,87]. Despite the great potential of this polymer, its poor stability over time renders chitosan-based systems not applicable as final pharmaceutical products. Therefore, scientists have put an effort into improving the stability characteristics of chitosan products. Several strategies have been proposed to preserve the initial properties of chitosan by preventing polymer chain damage (Figure 3).

### 4.1. Stabilizing Agents

As pharmaceutical products with chitosan are highly susceptible to physicochemical degradation upon storage, one of the goals for technologists is to apply the proper excipients in order to improve the chitosan-based system’s stability. It was previously explained that exposure to dry heat or steam sterilization has a marked effect on the properties and the end performance of the chitosan formulations [50,69,70,71,72,73]. Therefore, a number of stabilizing additives have been commonly tested in order to protect chitosan during thermal processing and/or sterilization treatment. Jarry *et al*., showed that the addition of polyols (mannitol, sorbitol, glycerol) to chitosan and chitosan/β-glycerophosphate solutions prior to autoclaving markedly slowed down polymer degradation [75]. In addition, the incorporation of polyol additives to chitosan solutions was found to have a protective effect on M_W,_ viscosity, and thermogelling properties. This phenomenon could be attributed to creation of a protective hydration layer around the chitosan chains through interchain hydrogen bonds. Luangtana-Anan *et al*., described the stability enhancement of chitosan microparticles prepared by ionotropic gelation and crosslinked with tripolyphosphate sodium in the presence of polyethylene glycol [94]. The addition of polyethylene glycol was shown to stabilize the zeta potential on the microparticles surface and prevent their aggregation over a period of one month. Conversely, the absence of the stabilizer resulted in a reduction in the particles’ electrostatic charge and led to aggregation after one-week of storage.

A possible destabilizing influence of the freeze-drying process may be overcome by the addition of disaccharides (such as mannitol, sucrose, and trehalose), which protect chitosan material from freezing stress [91]. However, due to the risk of Maillard reaction and colored products formation, reducing sugars (e.g., lactose, maltose) should not be considered as bioprotectans. The stabilizing effect of the sugars is explained by the fact that disaccharides act as water replacement agents interacting by hydrogen bonding, similarly to the replaced water. In addition, they form highly viscous sugar glasses which hinder the labile materials from disruptive reactions occurring upon freezing. Chitosan-DNA nanoparticles conjugated with polyethylene glycol at the nanoparticles surface could be successively lyophilized in the presence of mannitol. The dried particles were found not to aggregate and to be easily re-suspended in both saline and PBS, upon one-month storage at either 4 °C or −20 °C [95]. In another study with chitosan nanoparticles cross-linked with tripolyphosphate, Rampino *et al*., tested the influence of different bioprotectants—trehalose, mannitol and polyethylene glycol on the stability of the particles after drying by lyophilization or spray-drying technique [87]. The addition of trehalose to the suspension of nanoparticles significantly reduced particles aggregation enabling them to be re-dispersible after four-week storage and was the best protectant for both applied methods.

The addition of a plasticizer to chitosan films was found to influence the water-uptake and mechanical properties of chitosan formulations. Hermans *et al*., revealed that glycerol decreased the swelling ratio of ophthalmic chitosan formulations with cyclosporine A, and as a consequence prolonged and more controlled drug release profile was achieved [96]. However, no stability tests were provided in the study, thus it is difficult to predict the long-term effect of glycerol on the behavior of chitosan films upon storage [96]. Cervera *et al*., investigated the effect of different plasticizers—erythritol and glycerol—on the physical stability and sorption behavior of films prepared with chitosan and amylose corn starch blend [64]. The studies revealed the poor stability of films plasticized with erythritol as a result of liquefaction of the formulations in the presence of hygroscopic excipients—chitosan and erythritol. In contrast, composite films with glycerol were found to remain flexible and mechanically stable, although notable increase in water content upon three-month storage was observed.

Recently, metal ions have been used as agents which are able to increase the colloidal stability of chitosan polyelectrolyte complexes. Wu *et al*., showed that the size and polydispersity index of chitosan/hyaluronate complex remained stable in PBS suspension at room conditions over 35 days in the presence of zinc ions [97]. The mode of the Zn (II) stabilization effect could be attributed to formation of co-ordinate bonds that tune the morphology of the hyaluronate/chitosan complex followed by alteration of their swelling properties [97].

### 4.2. Chitosan Blends

In recent years, chitosan blends with nonionic polymers have received much attention because they are characterized by improved physicochemical properties in comparison to the pure polymer. In order to enhance the material stability, numerous studies have been reported on the binary mixtures of chitosan with both natural (starch) or synthetic poly(vinyl alcohol), poly(ethylene oxide), and polyvinylopyrrolidone polymers [56,64,98]. The specific interactions in the chitosan blends may involve hydrogen, ionic bonds, or dipole interference and final properties strongly depend on the miscibility of the components [99]. The films composed of binary mixtures of chitosan and amylose-corn starch plasticized with glycerol were found to be flexible and remained amorphous during three-month storage at 25 °C/60% RH and 40 °C/75% RH [64]. Studies conducted on the miscible blends with poly(vinyl alcohol) displayed a significant decrease in moisture sensitivity of chitosan [56]. The modification of the polymer structure using poly(vinyl alcohol) was noticed to limit its water-uptake proportionally with the concentration of synthetic polymer as a result of the increase in structural packing of chitosan [56]. In another study, Khoo *et al*., prepared homogenous chitosan/poly(ethylene oxide) and chitosan/polyvinylopyrrolidone films which were shown to possess higher initial temperature of thermal degradation compared to pure chitosan [98]. However, it should be noted that an improvement in thermal or hydrolytic stability might influence the biodegradability of chitosan blends which may become resistant to enzymatic degradation.

### 4.3. Chitosan Crosslinking

Chitosan modification through crosslinking is widely described in the literature and is a relatively easy method to prepare chitosan-based materials. On the basis of interaction between crosslinking agents and chitosan, chemical (covalent) and physical (ionic) crosslinking can be distinguished.

#### 4.3.1. Chemical Crosslinking

Chemical (covalent) crosslinking can effectively guard the physicochemical stability of chitosan applications since the gelation is irreversible. The higher stability of such modified chitosan is based on the covalent bonds, but also other interactions—hydrogen or hydrophobic bonds—cannot be excluded. To date, the most common chemical crosslinkers of chitosan are dialdehydes (such as glutaraldehyde or glyoxal [100,101,102]) and genipin [103]. However, chemical crosslinking also changes biological properties of chitosan material which may limit its practical use in pharmaceutical applications. In addition, dialdehydes are considered to be toxic, thus it is particularly important to completely eliminate the unreacted crosslinkers during the preparation process. The influence of chitosan structure modification by covalent crosslinking has been widely investigated [104], but only limited data have focused on the impact of these modifications on chitosan long-term stability. Liu *et al*., exhibited improved the physicochemical properties of chitosan/poly(acrylic acid) gel crosslinked with glutaraldehyde but the results were related only to freshly prepared formulations [105]. In another study, the stability of chitosan microspheres crosslinked with genipin in acidic conditions was investigated [103]. It was noted that the crosslinking level markedly influenced the swelling ability, mucoadhesiveness, and acidic stability of the prepared microparticles. However, besides improving the physicochemical properties, the crosslinking reaction between genipin and chitosan was found to be responsible for color alteration from transparent to blue [103,106]. Butler *et al*., revealed that the formation of blue pigments was a result of genipin polymerization induced by oxygen radicals [107]. As the presence of free radicals may also affect the chitosan structure, the above observations might be indicative of the impaired stability of chitosan/genipin materials upon storage under environmental conditions.

#### 4.3.2. Physical Crosslinking

In the ionic crosslinking process, a network of ionic bridges between negatively charged components and the positively charged chitosan chains is formed. Among ionic crosslinkers, small-size anions (as citrate, sulfate) or ionic molecules (e.g., phosphate-bearing groups) are commonly used. In addition, polyelectrolyte complexes (PEC) are included as a type of physical crosslinking, in which an additional natural or synthetic oppositely charged polymer is employed [108]. A list of the ionic crosslinkers commonly used for the modification of chitosan’s structure is presented in Table 4.

**Table 4:** Examples of ionic crosslinkers used for chitosan-based drug delivery systems and biomedical devices.

Type of the Ionic Crosslinker	Examples of Agents		
*metallic ions*	Fe(III)		
	Pt (II)		
	Mo(VI)		
*small-size anions* *or anionic molecules*	citric acid		
	succinic acid		
	sulfate sodium		
	inorganic phosphate salts	tripolyphosphate pentasodium	
		β-glycerophosphate disodium *	
		glucose-1-phosphate disodium *	
		glucose-6-phosphate disodium *	
*anionic polymer*	*natural*	carrageenan	
		gelatin	
		hyaluronic acid	
		kondagogu gum	
		pectin	
		γ-poly(glutamic acid)	
		sodium alginate	
		sodium dextran sulfate	
		xanthan gum	
	*synthetic*	poly(acrylic acid)	carbomer polycarbophil

		poly(methacrylate)	Eudragit
		poly(*N*-isopropylacrylamide)	poloxamer

*: the nature of interaction between polyol-phosphate agents and chitosan has not been clearly elucidated.

Physical modification of the chitosan structure, in contrast to chemical crosslinking, is a simple and mild process which requires neither the presence of catalysts nor the purification of the final product. The enhanced stability of the chitosan PEC can be attributed to the interaction between cationic chitosan and negatively charged complex polymer, which prevents the protonation of chitosan amino groups. In addition, the large anionic molecules are thought to buffer the solution and thus slow down the rate of chitosan hydrolysis.

However, chitosan crosslinked with small size ions is considered as unstable material over an extended time period because of the presence of the electrolytes and pH variations when stored in solution [109]. To our best knowledge, only a few attempts to improve long-term physicochemical stability of chitosan material by incorporation of the small-size ionic crosslinker have been reported. Chitosan co-crystals with acyclovir prepared by a solvent change method using sodium citrate as the salting out compound exhibited good physical stability with regard to the drug content and drug release profile upon three-month storage at 40 °C/75% RH [110]. Singh et al., studied the long-term stability of cisplatin-loaded chitosan glutamate microparticles, crosslinked with citric acid prepared by emulsification-ionotropic gelation [28]. No significant changes in physical appearance and drug content in all formulations stored in high-density polyethylene containers at 40 °C/75% RH and 25 °C/60% RH upon correspondingly 6- and 12-month periods were noticed. However, an approximately 17%–19% increase in moisture content and subsequent rise in the particle size during both long-term and accelerated stability studies were observed suggesting that chitosan microparticles might be still susceptible to physicochemical degradation over time [28].

Among a variety of chitosan applications, the stability of microparticulate-based delivery systems is extremely important as it strongly depends on the surface electrostatic charge, which alters upon storage. Several strategies have been proposed to prevent aggregation and changes in the zeta potential of chitosan micro- and nanoparticles. Insulin-loaded nanoparticles prepared by complexation of chitosan with dextran sulfate exhibited no significant differences in zeta potential and mean particle size up to 28 days at 4 °C [29]. Van der Lubben et al., demonstrated three-month physicochemical stability of chitosan microparticles crosslinked with sodium sulfate stored in PBS suspension under both refrigerated and ambient conditions [20]. No statistical differences in morphology and size of the particles, drug content and the drug release profile were displayed. Nevertheless, the improved stability of the microparticles could be attributed to the presence of the nonionic stabilizer—a polyoxyethylene sorbitan sodium monooleate (polysorbate)—used during preparation rather than the ionic crosslinking process. The potential stabilizing effect of freeze-drying step upon the microparticles elaboration process cannot be excluded as well. In another investigation, devoted to the polyelectrolyte complex nanoparticles composed of chitosan and hyaluronate, the authors noticed that prepared particles were stable in suspension up to four-week storage at room conditions. An increase in the amount of hyaluronate was found to be responsible for obtaining more stable formulations with minor fluctuations of zeta potential over storage period [111]. Mitra et al., revealed that introduction of succinic acid into chitosan/collagen PEC, notably improved the mechanical strength and thermal stability of the scaffold material [112].

A detailed stability analysis was also carried out on the chitosan/β-glycerophosphate (β-GP) *in situ* gels [113,114]. Those novel chitosan/polyol-phosphate compositions have gained great attention in the biomedical field due to the fact that modified chitosan becomes thermosensitive in diluted acids and can undergo gelation around body temperature [115]. These properties make chitosan/polyol-phosphate material a promising tool for a variety of applications, such as local drug delivery systems or injectable carriers for tissue-engineering. It should be noted that although the type of reaction between chitosan and polyol-phosphates is close to physical crosslinking, the nature of this interaction has not been clearly explained [27,116]. The fact that gelation appears even under refrigerated conditions is a substantial problem limiting thermoresponsive *in situ* chitosan/β-GP systems applications. Ruel-Gariepy *et al*., investigated the viscosity changes of chitosan/β-GP solutions upon three-month storage [117]. The studies revealed that the solution/gel transition appeared under both refrigerated as well as under room conditions [117]. Similarly, Schuetz *et al*., observed a gelation of the chitosan/β-GP within four-week storage at 4 °C confirming the instability of the systems under refrigerated conditions [93]. In contrast, the study on chitosan/β-GP gel formulation intended for periodontitis treatment—stored in closed containers at 30 °C/75% RH displayed no changes in color, consistency, pH, viscosity, and drug content over 90 days [113]. In order to overcome the poor stability of chitosan/β-GP thermogelling systems, Supper *et al*., proposed application of glycerol-1-phosphate as an alternative gelling agent for chitosan. The chitosan/glycerol-1-phosphate solutions were found to maintain their thermogelling properties for two months under ambient conditions and over nine months in a refrigerator [27].

Regardless of the above mentioned methods, it is extremely important to set-up the most suitable storage conditions which ensure sufficient stability of chitosan products. Hafner *et al*., in the long-term stability study of lyophilised lecithin/chitosan nanoparticles loaded with melatonin found that storage at 4 °C enabled their physicochemical properties to be retained without significant loss of encapsulated drug within a period of seven months [91]. The substantial changes in the rheological behavior of chitosan hydrogels with antifungal agent stored for a period of three months at 25 °C in comparison to hydrogels placed at 4 °C were reported [118]. A considerable (almost 50%) loss in viscosity values of the polymer-based formulation and a decrease in its pH at ambient temperature were found, whereas the physical stability of refrigerated formulations was shown not to statistically alter [118]. No *et al*., noticed the differences in viscosity of chitosan solutions at 4 °C and 25 °C after 15-week storage [78]. Although a drop of viscosity was more pronounced in solutions stored at ambient temperature, all chitosan formulations were characterized by weaker antibacterial activity compared to the antibacterial effect of freshly prepared solutions. However, Supper *et al*., indicated that the chitosan/β-GP thermogelling solutions intended for parenteral administration had poor physicochemical stability both under room and refrigerated conditions [114].

Selection of suitable humidity conditions is also important, especially for storage of solid chitosan-based products. Although no international requirements or standard references have been provided, several studies confirmed that the rate of hydration of chitosan products rose extensively at high RH [59,119]. Apart from selecting the most suitable storage conditions, proper air-tight containers, in order to protect hygroscopic chitosan products against environmental humidity should be also considered.

## 5. Conclusions

Despite the great potential of using chitosan in drug delivery or tissue engineering systems, its poor long-term stability is a substantial drawback in the scaling-up of chitosan pharmaceutical applications. Upon storage, chitosan undergoes gradual chain degradation followed by destruction of its functional groups which as a consequence leads to irreversible loss of its physicochemical properties. Both intrinsic (degree of deacetylation, molecular weight, purity, and moisture level) and extrinsic factors (environmental storage conditions, thermal processing, sterilization, and processing involving acidic dissolution) are acknowledged as crucial parameters affecting the stability of the chitosan-based formulations. To improve chitosan stability, several strategies (addition of the stabilizing agent during the preparation process, blending with hydrophilic polymer, and use of ionic or chemical crosslinkers) have also been reported. As there are no universal principles to preserve chitosan-based products upon storage, preformulation studies and selection of the most proper storage conditions are essential to provide their maximal stability.
